# A pragmatic randomised multi-centre trial of multifamily and single family therapy for adolescent anorexia nervosa

**DOI:** 10.1186/s12888-016-1129-6

**Published:** 2016-11-24

**Authors:** Ivan Eisler, Mima Simic, John Hodsoll, Eia Asen, Mark Berelowitz, Frances Connan, Gladys Ellis, Pippa Hugo, Ulrike Schmidt, Janet Treasure, Irene Yi, Sabine Landau

**Affiliations:** 1 Institute of Psychiatry, Psychology and Neuroscience, King’s College, London, London, UK; 2 Child and Adolescent Eating Disorders Service, South London and Maudsley NHS Foundation Trust, London, UK; 3 University College London, London, UK; 4 Marlborough Family Service, Central and North West London NHS Foundation Trust, London, UK; 5 Child and Adolescent Eating Disorders Service, Royal Free Hospital London, NHS Foundation Trust, London, UK; 6 Vincent Square Eating Disorders Service, Central and North West London NHS Foundation Trust, University of London, London, UK; 7 St George’s, University of London, London, UK; 8 Adult Eating Disorders Service, South London and Maudsley NHS Foundation Trust, London, UK; 9 Child and Adolescent Mental Health Service, Surrey and Borders Partnership NHS Foundation Trust, Frimley, UK

**Keywords:** Anorexia nervosa, Adolescents, Family therapy, Multi-family therapy, Randomised controlled trial

## Abstract

**Background:**

Considerable progress has been made in recent years in developing effective treatments for child and adolescent anorexia nervosa, with a general consensus in the field that eating disorders focussed family therapy (often referred to as Maudsley Family Therapy or Family Based Treatment) currently offers the most promising outcomes. Nevertheless, a significant number do not respond well and additional treatment developments are needed to improve outcomes. Multifamily therapy is a promising treatment that has attracted considerable interest and we report the results of the first randomised controlled trial of multifamily therapy for adolescent anorexia nervosa.

**Methods:**

The study was a pragmatic multicentre randomised controlled superiority trial comparing two outpatient eating disorder focussed family interventions - multifamily therapy (MFT-AN) and single family therapy (FT-AN). A total of 169 adolescents with a DSM-IV diagnosis of anorexia nervosa or eating disorder not otherwise specified (restricting type) were randomised to the two treatments using computer generated blocks of random sizes to ensure balanced numbers in the trial arms. Independent assessors, blind to the allocation, completed evaluations at baseline, 3 months, 12 months (end of treatment) and 18 months.

**Results:**

Both treatment groups showed clinically significant improvements with just under 60% achieving a good or intermediate outcome (on the Morgan-Russell scales) at the end of treatment in the FT-AN group and more than 75% in the MFT-AN group - a statistically significant benefit in favour of the multifamily intervention (OR = 2.55 95%; CI 1.17, 5.52; *p* = 0.019). At follow-up (18 months post baseline) there was relatively little change compared to end of treatment although the difference in primary outcome between the treatments was no longer statistically significant. Clinically significant gains in weight were accompanied by improvements in mood and eating disorder psychopathology. Approximately half the patients in FT-AN and nearly 60% of those in MFT-AN had started menstruating.

**Conclusions:**

This study confirms previous research findings demonstrating the effectiveness of eating disorder focused family therapy and highlights the additional benefits of bringing together groups of families that maximises the use of family resources and mutual support leading to improved outcomes.

**Trial Registration:**

Current Controlled Trials ISRCTN11275465; Registered 29 January 2007 (retrospectively registered)

## Background

In the past 10–15 years there has been considerable progress in the evaluation of treatments for child and adolescent anorexia nervosa (AN). In spite of this, the gaps in our knowledge are still considerable. The only treatment with significant support from randomised controlled trials (RCTs) is family therapy (FT) [1–5]. Four studies have compared FT with individual therapy or treatment as usual either as follow-up to inpatient treatment [6, 7] or as stand-alone outpatient treatments [8, 9] showing positive benefits for FT. Three studies have compared different forms of family therapy [10, 11] or different lengths of treatment of the same therapy [12]. Four of the above studies [13–16] have completed follow-ups of between 3–5 years showing low relapse rates (of less than 10%) and continued improvements post treatment. With one exception all the studies have used an anorexia nervosa focused family therapy (FT-AN).^1^

Godart et al. [6] evaluated a FT that did not have an eating disorders focus and still showed positive benefit compared to treatment as usual. Agras et al. [11] compared FT-AN with a generic systemic FT showing generally similar outcomes between the two treatments but with some advantages for FT-AN in faster weight gain early on in treatment, fewer days in hospital if admission was needed and lower costs. While many of the earlier studies had relatively small samples, the two most recent studies [8, 11] had adequate power and confirm the findings from earlier studies. Two individual treatments have some support. The studies by Robin et al. [9] and Lock et al. [8] used the same individual therapy (called Ego Oriented Individual Therapy in the former and Adolescent Focussed Therapy in the latter), which while somewhat less efficacious than family therapy, nevertheless, lead to a good outcome for a significant number of patients. There is also some evidence in support of CBT though there has only been one very small RCT comparing CBT with FT showing no difference in outcome [17] and an open follow up study [18], which showed a positive treatment response in those who completed the full 40 week treatment (just under two thirds of the sample) and ongoing improvements over the 60 week follow-up. Apart from a somewhat lower retention rate in treatment compared to most of the FT-AN studies reported above, the CBT treatment was also more intensive, requiring approximately twice the number of sessions then is usually the case with FT-AN.

While the outcome results of FT-AN are encouraging, not everyone responds, with 10–20% requiring additional, more intensive treatment (medical stabilization in hospital, inpatient psychiatric treatment or day care/partial hospitalization) and 10–15% continue to need ongoing treatment into adulthood [19]. In recent years there has therefore been growing interest in modifying or enhancing FT-AN that some families might need to gain further benefit. These have included adding a focus on addressing difficulties with emotional issues and attachment patterns in the family [20, 21], further strengthening parental management of feeding the young person [22–24], and also bringing together groups of families in a multi-family therapy context [25–27].

Multi family therapy for anorexia nervosa (MFT-AN) draws on the same principles as FT-AN but is delivered in a more intensive format to help families to overcome a sense of isolation and stigmatization and to maximize their own resources. Generally between 5–7 families take part in MFT, sharing their experiences, learning by example and learning from and providing support for one another. In MFT-AN the principles of FT-AN [19, 28–30] are integrated with more general concepts of MFT [25–27, 31] as well a broad range of cognitive, psychodynamic and group therapy conceptualizations and intervention techniques. Several descriptive and open follow-up studies have reported high levels of satisfaction with the treatment and minimal dropouts [26, 32], improvements in symptoms and interpersonal functioning [32–35] and reductions in hospital readmission rates [36]. In this paper we describe the first RCT of MFT-AN comparing its relative effectiveness with FT-AN. Our primary hypothesis was that MFT-AN would be more effective than FT-AN in restoring the young person to a healthy nutritional state. A subsidiary hypothesis was that those participating in MFT-AN would express higher satisfaction with treatment compared to those allocated to FT-AN.

## Method

### Participants and the context of the study

The study was a pragmatic multicentre randomised controlled superiority trial comparing MFT-AN with FT-AN. The trial was carried out in six specialist eating disorders services in the National Health Service in the UK providing treatment in a defined catchment area in or near London. Patients invited to take part in the study were consecutive referrals to the services, who met the inclusion criteria. To be eligible for the study patients had to be aged between 13-20 years,^2^ fulfil DSM-IV criteria for anorexia nervosa or eating disorders not otherwise specified (restricting type) and either be below 86% median BMI (%mBMI) for age and sex or had lost 15% body weight in the last three months. Patients were excluded if they were in care, had a learning disability, were suffering from psychosis, were alcohol or substance dependent or had a coexisting medical condition that might impact their weight (such as diabetes). We also excluded patients who were seriously medically at risk that is having a weight less than 67% mBMI or meeting one or more criteria for medical instability eg dehydration (a postural drop of 20 mmHg), bradycardia (pulse rate below 40 BPM), failed squat test (unable to get up without using arms as levers),  temperature below 34.5 °C or electrolyte imbalance.

Therapists delivering the treatment were not selected specifically for the study but included all the clinicians routinely providing therapy in the participating services (including trainees in the service). Training in both treatments was provided by two of the study authors (IE, EA) at the start of the study and regular six monthly meetings of all trial therapists were used to ensure consistency of approach across the centres was maintained. Within each service regular supervision was provided for each treatment by experienced supervisors. Both treatments are manualized [37].

To ensure that families did not have to wait unnecessarily long before being enrolled in a group the MFT groups rotated between participating centres and were comprised of families from across the centres. Groups consisted of 5–7 families and could also include non-trial families. Typically a group would contain 3–5 trial families. Two therapists always provided treatment with the therapist pairings changing from group to group.

### Trial registration and ethical approval

The trial was registered with Current Controlled Trials (registration number ISRCTN11275465) and with the UK Clinical Research Network (UKCRN ID 6041). Ethical approval for the study was given by the UK Integrated Research Approval System Committee (04/MREC/022). All participants gave written informed consent having been provided details of the study and clear information that deciding not to take part in the study or withdrawing from the study at any point would have no prejudicial effect on the treatment offered to them by the participating services. For those under the age of 16, consent was obtained from the young person’s parents or legal guardians.

### Treatments

#### Outpatient family therapy

Family therapy for adolescent anorexia nervosa (FT-AN) [19, 29, 31] has been the focus of our previous treatment trials [7, 10, 13, 38] – see also Eisler et al. [30] for an account of the evolution of the treatment) and a treatment manual has been developed to guide the therapists’ interventions [37]. Patients are seen with their families over a period of 12 months. The number and frequency of sessions is determined by clinical need starting initially with weekly meetings, which are then gradually spread out to 3–4 weekly. These are mainly conjoint family meetings although some individual sessions are included where appropriate (particularly with older adolescents at later stages of the treatment). The treatment approach has the following key features:

- A systems focus on understanding the family in the context of a potentially life threatening illness where the family is needed as a resource to help their child recover, emphasizing that the family is not the cause of the illness.
- An emphasis on helping the parents to take a lead in managing their child’s eating in the early stages of treatment whilst stressing the temporary nature of this role and making clear that this is an expression of parental care rather than parental control.
- Psychoeducation about the effects of starvation and the role of predisposing personality traits for the development of anorexia nervosa and comorbidities.
- Use of externalisation of the illness using a range of techniques including narrative externalising conversations [39] that implies anorexia is a separate entity, as well as discussions of the psychological and physiological effects of starvation, which are used purposefully to emphasize the role that starvation has in influencing both behaviours and cognitions, thereby shaping and reinforcing the illness process.
- In later stages of treatment a shifting of focus on adolescent and family developmental life cycle issues. The focus in this later stage is to help the family to disentangle individual psychological issues (eg self esteem, individuation, psychosocial functioning) and family relationship issues from the eating disorder behaviour and the interactional patterns that have developed around it.

#### Multi-family therapy

Multi-family therapy for anorexia nervosa (MFT) [27, 31] shares the main conceptual principles with FT-AN but provides a more intensive form of treatment and allows the family to draw on support from other families in the group. The treatment starts with an intensive four day multi-family programme for 5–7 families and is followed by a further six one day meetings at 4–8 week intervals over 9 months. Individual family meetings are scheduled in the intervals between group meetings as needed with the overall length of treatment for each family being 12 months. A wide range of intervention techniques is used (including group, family, psycho-educational and creative techniques) combining multi-family sessions with separate parent and adolescent sessions as well as activities targeted at individual families. There is also practical input around managing mealtimes with multifamily meals and ongoing discussions of what works best for each family in how parents help their child overcome the fear of eating and gaining weight.

In addition to the general principles of FT-AN, described above, MFT-AN includes the following [25, 27]:

- Creating a sense of solidarity and reducing social isolation and stigmatisation.
- Stimulating new perspectives and providing a context where families learn from each other.
- Strengthening self-reflectiveness through observing others, encouraging mutual support and feedback and experimenting with cross-family exercises.
- Discovering and building on competencies, intensifying interactions and experiences and practicing new behaviours in a safe space.
- Raising expectations and hopes for recovery.

Families randomised to the MFT-AN group were all initially engaged in treatment individually until 5–7 families had been recruited to create a new group (a new group thereby starting approximately every six weeks). Families were seen in between group meetings, the frequency and overall number of such meetings determined by clinical need although the expectation was that these sessions would be less frequent than in the FT-AN group.

### Assessments

Independent research assessors, who were blind to the treatment allocations, conducted evaluations pre-randomisation (baseline), at 3 months, 12 months (end of treatment) and 18 months (6 month post treatment). Every effort was made to include all patients in the follow-up assessments, including those who discontinued treatment early, to allow for an intention to treat analysis.

Primary outcome was defined using the Morgan/Russell Global outcome scale [40] modified by Russell et al. [7] to take account of bulimic symptoms which provides three categories of outcome: *Good outcome* includes patients whose weight is above 85% mBMI, who are menstruating and have no bulimic symptoms. Those in the *intermediate outcome* group meet the same weight criteria but are either not menstruating or have occasional bulimic symptoms (averaging less than once a week over the past month). Patients whose weight is below 85% mBMI or have developed bulimic symptoms of once a week or more are classified as having a *poor outcome*. The primary time point of interest was the end of treatment (12 months post randomisation).

Secondary outcomes of interest at 12 months and 18 months post randomisation were:

- Morgan/Russell Global outcome scale at 18 months.
- Weight data reported as a percentage of median (ie 50^th^ centile) BMI for young people of the same height, age and sex, which takes into account that BMI in children and adolescents changes with age. The conversions to %mBMI were done using a computer programme based on the Child Growth Foundation [41] development charts.
- The Eating Disorder Examination [42], a well validated interview measure of eating disorder psychopathology, was administered at baseline, 12 months and 18 months. In addition to a global scale there are 4 subscales (Restraint; Eating Concern; Shape Concern and Weight Concern).
- Self-report measures completed by the young people (administered at all four time points) included:
  - o Beck Depression Inventory [43]
  - o Rosenberg Self-Esteem Scale [44]
- Parents completed the Experience of Caregiving Inventory [45] (at baseline and 12 months) a measure developed for the assessment of caregiving demands with severely mentally ill patients adapted for use in eating disorders [46].
- All participants also completed the Client Satisfaction Questionnaire [47] at the end of treatment.

### Randomisation

Random allocations of patients to trial arms at a 50:50 ratio were computer generated. Randomisation was conducted in blocks of random sizes between 6 and 12 patients to ensure equal numbers of patients in the trial arms. Once the initial assessment was carried out and the patient was recruited to the trial, the trial co-ordinator allocated the patient immediately to one of the treatment groups using a randomisation database created before the beginning of the study. This database returned a patient's random group allocation once the patient ID was entered and did not allow any changes to the group allocation thereafter. The research workers carrying out the assessments were blind to the allocation. The trial co-ordinator informed the therapist assigned to the case who then contacted the family to arrange the start of the allocated treatment.

### Sample size calculation

For outpatients taking part in FT-AN we expected 63% to reach a weight that is above 85% mBMI at the end of treatment (12 months) based on Eisler et al. [10] and Robin et al. [48]. Weight gain during MFT-AN had not been studied in as much detail but our preliminary data [26] suggested that 83% or more patients could be expected to have reached a healthy weight within the same period. We calculated that 77 participants would be needed per trial arm for a two-sided chi-squared test to detect such a difference with 80% power at a 5% significance level. Allowing for 15% attrition to follow-up required raising the number to 91 per trial arm (a total sample size of *n* = 182).

### Statistical analysis

All formal statistical analyses were based on an intent-to-treat principle with all participants analysed in the treatment arm to which they were assigned. The primary outcome was a binary variable contrasting good or intermediate versus a poor outcome on the Morgan Russell Global scale at the end of treatment, 12 months post-randomisation, analysed using logistic regression. The model contained trial arm, baseline status on the Morgan Russell scale (binary variable), whether the patient had previous treatment for eating disorder and time (months) since diagnosis of the eating disorder as explanatory variables. The latter three baseline variables were included to gain precision since they were known predictors of outcome. We report inferences for the odds ratio of good/intermediate outcome comparing MFT-AN with FT-AN at 12 months. Odds presented are marginal population odds derived from the (conditional) subject specific OR by an approximate conversion formula [49]. We present marginal odds as representing the group difference in average treatment effects on the population of ED patients (as opposed to the difference in individual change across time described by conditional OR [49]).

Good or intermediate versus a poor outcome on the Morgan Russell scale at 18 months post-randomisation was also analysed using logistic regression. Other continuous secondary outcomes were analysed using analogous linear regression models. Normality of error terms was checked using residual diagnostics.

To investigate drivers of loss-to-follow up in primary and secondary outcomes, an indicator of missingness at 12 month was generated for each outcome variable. Then logistic regressions were used to assess whether there were any associations between baseline variables or treatment adherence and missingness. Specifically, the logistic regression model contained trial arm as a predictor and added baseline variables and engagement in treatment in a forward fashion to assess their predictive power; with variables showing an association at the liberal 15% level considered potential missingness predictors. For this purpose engagement in treatment was measured by a binary variable indicating whether or not the family attended the full 12 months of treatment. As incomplete engagement with treatment was found to be predictive of missingness, we employed multiple imputation (MI, with 100 imputations) which enabled us to generate inferences that are valid under a missing at random assumption (MAR) with the empirically identified variables (including post-randomisation treatment engagement) being allowed to predict missingness [50]. Specifically the imputation step included all the variables of the respective analyses model, further measures of the outcome variable at other assessment time points and the predictors of missingness.

Finally differences in carer and young person satisfaction were investigated using regression of the CSQ difference scores between carer and young person. A simple regression model testing whether the intercept (of the difference scores) was different from 0 was used in conjunction with MI. The statistical significance level was set to 5%. All statistical analyses were carried out in Stata 14.1 and R 3.2 (figures were produced with ggplot2 [51]).

## Results

### Participant characteristics

**Fig. 1:**
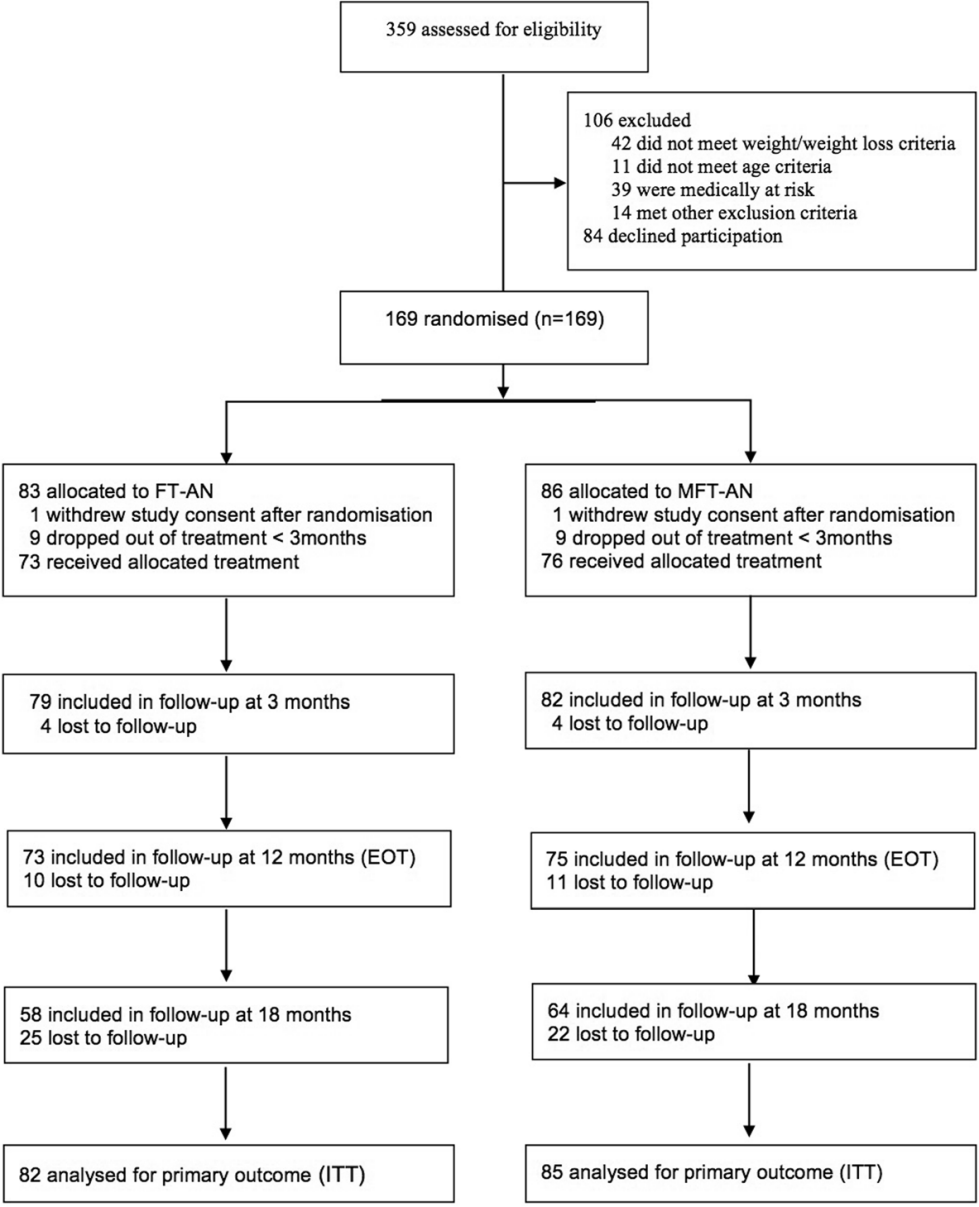
CONSORT Diagram

Figure 1 shows the CONSORT diagram for the study - 359 adolescents were screened; 253 met the inclusion criteria and 169 agreeing to take part in the study. Two patients who agreed to participate and were randomised, subsequently withdrew consent and did not contribute to the analysis sample. A total of 167 adolescents took part in the study (82 in FT-AN and 85 in MFT-AN). Table 1 shows the participant demographic and clinical characteristics. The mean age on entry to the study was 15.7 (SD 1.7), 91% were female, 70% came from intact families and just under 10% had a non-white ethnic background. The mean %mBMI on entry was 78.0 (SD 6.1) and the median duration of illness was 7 months. Seventy six percent met diagnostic criteria for anorexia nervosa the remaining were classified as EDNOS (restricting type). Table 1 also shows that randomisation produced reasonably balanced group with none of the observed differences between the treatment groups reaching statistical significance.

**Table 1:** Baseline demographic and clinical factors

	FT - AN (N = 82)	MFT - AN (N = 85)
Demographics		
Age: mean (sd)	15.7 (1.6)	15.7 (1.7)
Female: n (%)	77 (93.9)	75 (88.2)
Ethnicity: n (%)		
White	76 (92.7)	75 (88.2)
Other	3 (3.7)	3 (3.5)
Missing	3 (3.7)	7 (8.2)
Education: n (%)		
Pre GCSE	54 (65.9)	60 (70.6)
Post GCSE	26 (31.7)	22 (25.9)
Missing	2 (2.4)	3 (3.5)
Parents: n (%)		
Married / Cohabit	56 (68.3)	61 (71.8)
Separated / Divorced	24 (29.3)	24 (28.2)
One Parent Deceased	2 (2.4)	0 (0)
Clinical Characteristics		
ED Age of onset (years): mean (SD)	14.3 (1.8)	14.5 (1.7)
Missing: n (%)	6 (7.3)	12 (14.1)
ED Duration (months): Median (IQR)	7 (4 – 11.5)	7 (3 – 11)
^a^Mean (SD)	11.4 (12.6)	9.6 (10.5)
Missing: n (%)	6 (7.3)	12 (14.1)
Eating disorder: n (%)		
Anorexia Nervosa	67 (81.7)	60 (70.6)
EDNOS (Restricting)	15 (18.3)	25 (29.4)
Prior Inpatient Treatment: n (%)		
Yes	16 (19.5)	11 (12.9)
No	54 (66%)	57 (67%)
Missing	12 (14.6)	17 (20.0)
Co-morbid Depression: n (%)		
Yes	10 (12.2)	2 (2.4)
No	57 (69.5)	66 (77.7)
Missing	15 (18.3)	17 (20.0)
Co-morbid OCD: n (%)		
Yes	3 (3.7)	0 (0.0)
No	64 (78.1)	68 (80.0)
Missing	15 (18.3)	17 (20.0)
Co-morbid Anxiety: n (%)		
Yes	1 (1.2)	0 (0.0)
No	66 (80.5)	68 (80.0)
Missing	15 (18.3)	17 (20.0)
Outcomes at baseline		
%mBMI: mean (SD)	78.4 (5.8)	77.6 (6.3)
BMI: mean (SD)	15.8 (1.2)	15.7 (1.3)
EDE (restraint): mean (SD)	2.9 (1.7)	2.7 (1.8)
Missing: n (%)	10 (12.2)	10 (11.8)
EDE (eating concern): mean (SD)	2.1 (1.5)	1.9 (1.6)
Missing: n (%)	10 (12.2)	10 (11.8)
EDE (shape concern): mean (SD)	3.2 (1.9)	2.9 (1.8)
Missing: n (%)	10 (12.2)	10 (11.8)
EDE (weight concern): mean (SD)	2.8 (1.9)	2.5 (1.8)
Missing: n (%)	10 (12.2)	10 (11.8)
BDI: mean (SD)	25.2 (14.4)	23.9 (14.3)
Missing: n (%)	20 (24.4)	16 (18.8)
RSE: mean (SD)	26.3 (6.9)	25.1 (6.6)
Missing: n (%)	19 (23.2)	17 (20)

*Abbreviations*: %*mBMI* a percentage of median Body Mass Index for young people of the same height, age and sex, *EDE* Eating Disorder Examination, *BDI* Beck’s Depression Inventory, *RSE* Rosenberg Self Esteem Scale

^a^because the duration of illness distribution is skewed, the median is the appropriate measure of central tendency; most other studies have used the mean (sd) value and we report this as well to provide comparison with other reports

### Engagement in treatment

Engagement with treatment was high with only 9 families in each group discontinuing treatment early (defined as attending for < 3 months) ie less than 11% of the total. We had hypothesised that the addition of the MFT sessions would reduce the number of outpatient sessions required but while the median number of outpatient sessions attended in the MFT-AN group was lower than in the FT-AN group (median18.5; IQR 11–24 compared to 19; IQR 12 - 27) the difference was not statistically significant (Mann–Whitney test of group difference z = 0.99, *p* = 0.32). In addition to the single family therapy sessions those in the MFT-AN group attended a median 7 MFT days; IQR 1 – 10.

### Loss to follow-up

There was little attrition with regard to the primary Morgan Russell outcome and %mBMI (19 and 20 values at end of treatment and 6 months post treatment respectively) but loss to follow-up was considerable for secondary outcome measures (median 88 values or 53% missing). A number of baseline variables predicted missingness (family history of eating disorder, co-morbidity and birth order in family). In particular not completing full 12 months of treatment was found to be a clear predictor of missing outcome data on the Morgan Russell scale (OR 12.5, 95% CI: 1.49 to 104.9 z = −2.3, *p* = 0.02). We therefore employed multiple imputation to generate results that are valid under less restrictive assumptions that allowed for these variables to predict missingness (see Methods).

### Categorised clinical outcome over the course of treatment and six month follow-up

**Fig. 2:**
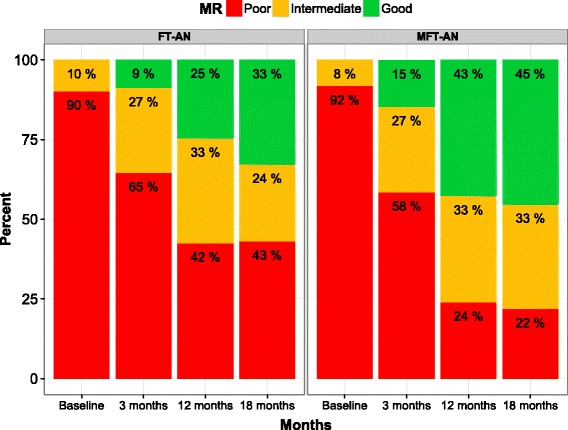
Distribution of Morgan Russell categorisation by trial arm

Figure 2 summarises the raw categorised outcome in the two treatment groups at 3 months, 12 months (end of treatment) and 18 months (six-month follow-up). Table 2 shows a statistically significant difference in the primary outcome (rating on the Morgan-Russell Global outcome scale at 12 months) in favour of MFT-AN with an odds of a good or intermediate outcome in the MFT-AN group 2.55 times higher than that of FT-AN (*p* = 0.018; 95% CI 1.17 to 5.52; p = 0.018). At 18 month follow-up (secondary outcome) the odds ratio had dropped to 2.01 and this weaker effect was not significant (95% CI 0.91 to 4.45; *p* = 0.086). The distribution of the outcome findings was similar across the different treatment centres.

**Table 2:** Estimated mean outcome differences between treatment arms at end of treatment and follow-up

	End of treatment (12 months post randomisation)			Follow-up (18 months post randomisation)		
	Mean group difference (95% CI)	Test	Standardised Coefficient	Mean group difference (95% CI)	Test	Standardised Coefficient
Binary MR	2.55^a^ (1.17, 5.52)	t = 2.36 *p* = 0.018	-	2.01^a^ (0.91, 4.49)	*t* = *1.72* *p* = *0.086*	-
%mBMI	2.24 (−0.47, 4.95)	*t = 1.62* *p* = *0.105*	0.37^b^	4.11 (0.98, 7.24)	*t = 2.57* *p* = *0.010*	0.68^b^
BMI	0.40 (−0.15, 0.94)	*t = 1.43* *p* = *0.15*	0.37^b^	0.74 (0.12, 1.36)	*t = 2.35* *p* = *0.019*	0.60^b^
EDE (restraint)	0.45 (−0.22, 1.11)	*t = 1.33* *p* = *0.185*	0.26^b^	0.45 (−0.26, 1.15)	*t =1.25* *p* = *0.213*	0.26^b^
EDE (eating concerns)	0.14 (−0.46, 0.73)	*t = 0.44* *p* = *0.659*	0.087^b^	0.18 (−0.42, 0.78)	*t = 0.59* *p* = *0.559*	0.12^b^
EDE (shape concerns)	0.54 (−0.18, 1.26)	*t =1.48* *p* = *0.139*	0.30^b^	0.56 (−0.20, 1.31)	*t = 1.44* *p* = *0.150*	0.30^b^
EDE (weight concerns)	0.44 (−0.27, 1.14)	*t = 1.22* *p* = *0.223*	0.23^b^	0.49 (−0.31, 1.29)	*t =1.21* *p* = *0.226*	0.26^b^
BDI	2.91 (−3.10, 8.91)	*t = 0.95* *p* = *0.342*	0.20^b^	2.14 (−4.16, 8.44)	*t = 0.67* *p* = *0.504*	0.15^b^
RSE	−1.09 (−3.91, 1.73)	*t = −0.76* *p0.447*	−0.16^b^	0.5 (−2.45, 3.45)	*t = 0.33* *p* = *0.739*	0.08^b^
ECI - Negative	−2.22 (−17.1, 12.6)	*t = −0.30* *p* = *0.766*	−0.07^b^	-	-	-
ECI - Positive	0.71 (−2.07, 3.49)	*t = 0.51* *p* = *0.612*	0.08^b^	-	-	-
CSQ - Young Person	−1.14 (−3.73, 1.45)	*t = −0.88* *p* = *0.382*	−0.22^c^	-	-	-
CSQ - Parent	0.64 (−1.51, 2.79)	*t = 0.5962* *p* = *0.556*	0.13^c^	-	-	-

Results are derived using multiple imputation with 100 imputations

*Abbreviations*: *MR* Morgan Russell Scale, *%mBMI* a percentage of median Body Mass Index for young people of the same height, age and sex, *EDE* Eating Disorder Examination, *BDI* Beck Depression Inventory, *RSE* Rosenberg Self Esteem Scale, *ECI* Experience of Caregiving Inventory, *CSQ* Client Satisfaction Questionnaire

^a^the effect is expressed as a marginal odds ratio (OR); The conditional OR was 4.57 (95% CI 1.28, 16.44) at 12 m and 3.16 (95% CI 0.84, 11.82) 18 m

^b^standardised coefficients were derived from dividing estimated difference by the standard deviation of the outcome variable at baseline

^c^standardised coefficients were derived from dividing difference scores by the standard deviation of the control group (FT-AN)

### Changes in weight, eating disorder psychopathology, depression and self-esteem

**Fig. 3:**
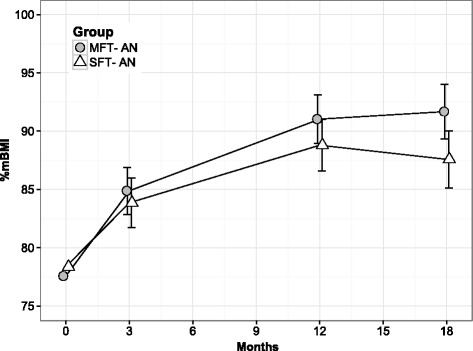
Estimated weight gain over the course of treatment and follow-up. Symbols represent estimated means and error bars associated 95% confidence intervals. Estimated means represent patients with the baseline mean %mBMI, mean time of eating disorder in months, no previous treatment, no family history of eating disorder and eldest of birth order in family

The adolescents in both treatment groups gained considerable weight over the course of treatment and follow-up (Fig. 3). Table 2 shows the results of the trial arm comparisons at 12 months and 18 months and Table 3 provides estimated changes in either trial arm since baseline. At 12 months there were no statistically significant differences between the two trial arms for %mBMI, eating disorder psychopathology, depression or self-esteem (Table 2). At 18 months there was a significant difference in %mBMI favouring MFT-AN (difference = 4.11; 95% CI 0.98, 7.24; *p* = 0.01). There were no significant differences on any of the other measures.

**Table 3:** Estimated mean change^a^ from baseline at end of treatment (12 months) and follow-up (18 months)

	End of treatment (12 months post randomisation)						Follow-up (18 months post randomisation)				
	Group	Observed: n (%)	Mean Change (95% CI)	Test	Standardised Coefficient	12m Predicted Mean (95% CI)	Observed: n (%)	Mean Change (95% CI)	Test	Standardised Coefficient^b^	18 m predicted mean (95% CI)
%mBMI Baseline = 77.95	FT-AN	72 (88%)	10.84 (8.64, 13.04)	*t = 9.66* *p < 0.001*	1.79	88.78 (86.59, 90.98)	55 (67%)	9.63 (7.18, 12.08)	*t = 7.71* *p < 0.001*	1.59	87.58 (85.13, 90.03)
	MFT-AN	75 (88%)	13.08 (10.98, 15.18)	*t = 12.22* *p < 0.001*	2.16	91.03 (88.94, 91.13)	60 (71%)	13.74 (11.41, 16.08)	*t = 11.53* *p < 0.001*	2.27	91.68 (89.33, 94.03)
BMI Baseline = 15.7	FT-AN	72 (88%)	2.61 (2.16, 3.06)	*t = 11.31* *p < 0.001*	2.09	18.31 (17.86, 18.76)	55 (67%)	2.59 (2.10, 3.08)	*t =10.39* *p < 0.001*	2.07	18.29 (17.80, 18.78)
	MFT-AN	75 (88%)	3.01 (2.59, 3.42)	*t =14.14* *p < 0.001*	2.41	18.71 (18.29, 19.12)	60 (71%)	3.33 (2.87, 3.79)	*t = 14.24 p < 0.001*	2.66	19.03 (18.57, 19.49)
EDE (restraint) Baseline = 2.78	FT-AN	38 (46%)	−1.38 (−1.90, −0.86)	*t = −5.23* *p < 0.001*	−0.80	1.39 (0.88, 1.92)	32 (39%)	−1.59 (−2.12 −1.04)	*t = −5.77* *p < 0.001*	−0.91	1.20 (0.66, 1.74)
	MFT-AN	44 (52%)	−0.93 (−1.42 −0.44)	*t = −3.76* *p* = *0.005*	−0.52	1.85 (1.36, 2.34)	38 (45%)	−1.14 (−1.64 −0.63)	*t = −4.45* *p < 0.001*	−0.75	1.64 (1.14, 2.15)
EDE (eating concerns) Baseline = 2.01	FT-AN	38 (46%)	−0.83 (−1.30 −0.35)	*t = −3.46* *p < 0.001*	−0.54	1.18 (0.71, 1.65)	32 (39%)	−1.10 (−1.57 −0.64)	*t = −4.25* *p < 0.001*	−0.72	0.91 (0.44, 1.37)
	MFT-AN	44 (52%)	−0.70 (−1.13 −0.25)	*t = −3.10* *p* = *0.002*	−0.45	1.32 (0.88, 1.76)	38 (45%)	−0.92 (−1.35 −0.50)	*t = −4.25* *p < 0.001*	−0.605	1.09 (0.66, 1.51)
EDE (shape concerns) Baseline = 1.83	FT-AN	37 (45%)	−1.28 (−1.85 −0.71)	*t = −4.44* *p < 0.001*	−0.70	1.75 (1.18, 2.32)	32 (39%)	−1.25 (−1.84 −0.66)	*t = −4.16* *p < 0.001*	−0.68	1.78 (1.19, 2.37)
	MFT-AN	44 (52%)	−0.74 (−1.28 −0.20)	*t = −2.70* *p* = *0.0072*	−0.41	2.29 (1.75, 2.82)	38 (45%)	−0.69 (−1.24 −0.15)	*t = −2.491* *p < 0.013*	−0.38	2.34 (1.79, 2.88)
EDE (weight concerns) Baseline = 2.64	FT-AN	38 (46%)	−1.03 (−1.57 −0.48)	*t = −3.67* *p < 0.001*	−0.54	1.62 (1.07, 2.16)	31 (38%)	−0.88 (−1.50 −0.26)	*t = −2.80* *p* = *0.006*	−0.47	1.76 (1.14, 2.38)
	MFT-AN	44 (52%)	−0.59 (−1.12 −0.06)	*t = −2.18* *p* = *0.029*	−0.31	2.05 (1.52, 2.58)	37 (44%)	−0.39 (−0.96, 0.18)	*t = −1.34* *p* = *0.181*	−0.21	2.25 (1.68, 2.82)
BDI Baseline = 24.47	FT-AN	35 (43%)	−8.82 (−13.31 −4.33)	*t = −3.86* *p < 0.001*	−0.62	15.65 (11.2, 20.14)	37 (45%)	−7.40 (−12.04 −2.75)	*t = - 3.13* *p* = *0.002*	−0.52	17.07 (12.43, 21.72)
	MFT-AN	41 (48%)	−5.91 (−10.20 −1.63	*t = −2.71* *p* = *0.007*	−0.41	18.55 (14.27, 22.84)	33 (39%)	−5.26 (−9.89 −0.62)	*t = −2.23* *p* = *0.026*	−0.37	19.22 (14.6, 23.85)
RSE Baseline = 25.68	FT-AN	36 (44%)	−0.51 (-2.74, 1.72)	*t = −0.45* *p* = *0.653*	–0.08	25.19 (22.96, 27.42)	37 (45%)	–1.50 (–3.80, 0.79)	*t = –1.29* *p* = *0.199*	–0.51	24.20 (21.90, 26.49)
	MFT-AN	42 (49%)	–1.60 (–3.65, 0.44)	*t =–1.54* *p* = *0.124*	–0.24	24.10 (22.05, 26.14)	35 (41%)	–1.00 (–3.11, 1.11)	*t = –0.93* *p* = *0.352*	–0.15	24.70 (22.58, 26.18)
ECI (Negative) Baseline = 85.56	FT-AN	44 (54%)	–13.59 (–24.58,–2.59)	*t = −2.47* *p = 0.016*	−0.45	71.97 (60.98, 82.97)					
	MFT-AN	44 (52%)	−15.81 (−26.88 −4.72)	*t = −2.86* *p = 0.006*	−0.52	69.75 (58.68, 80.83)					
ECI (Positive) Baseline = 28.27	FT-AN	47 (57%)	0.42 (−1.61, 2.45)	*t−0.41* *p = 0.682*	0.05	28.67 (26.66, 30.72)					
	MFT-AN	46 (54%)	1.13 (−0.99, 3.25)	*t−1.07* *p = 0.291*	0.13	29.40 (27.28, 31.52)					

The number of observations is presented with percentage of observations possible in each treatment arm. Estimates are derived using multiple imputation with100 imputations

*Abbreviations*: *%mBMI* a percentage of median Body Mass Index for young people of the same height, age and sex, *EDE* Eating Disorder Examination, *BDI* Beck Depression Inventory, *RSE* Rosenberg Self Esteem Scale, *ECI* Experience of Caregiving Inventory

^a^To derive predicted mean change scores, continuous covariates were set at the mean of the sample at baseline and categorical values at the most frequent value: For each outcome, at the baseline mean of that outcome and the following additional estimates: Eating disorder in months; mean 9.34, previous Eating Disorder patient: no, family history of Eating Disorder: birth order in family; eldest

^b^standardised coefficients were calculated by dividing estimated mean change by the standard deviation of the outcome variable at baseline

The increase in weight resulted that in FT-AN 51.2% had started menstruating at 12 months (not including boys and those taking oral contraception) and 51.4% 18 months. In the MFT-AN group the figures were 56.3% at 12 months and 59% at 18 months.

A small number of the young people had developed some bulimic symptoms – in FT-AN three at 12 months and two at 18 months, in MFT-AN five (one of whom would have met diagnostic criteria for bulimia nervosa) at 12 months and three at 18 months.

There were significant improvements over time in eating disorders psychopathology and depression in both groups (Table 3). There were only small and non-significant improvements in ratings of self-esteem over time (Table 3) although it should be noted that the average scores on this measure were in the normal range already at baseline (Table 1).

### Admissions to hospital during the study

A small number of patients needed to be admitted to hospital during the course of treatment, three in the in the MFT-AN group (2 ED related; 1 because of suicidal risk) and two in the FT-AN group (both ED related). Following discharge from hospital the patients continued with their outpatient treatment until the planned end of treatment at 12 months. During the six month follow-up period after the end of the planned outpatient treatment there were six further admissions (2 in the MFT-AN arm and 4 in the FT-AN arm) – all six patients had made poor progress in outpatient therapy and had been categorised as having a poor outcome at 12 months

### Satisfaction with treatment

Data related to treatment satisfaction can be found in Table 4. It is important to note that only 60% of parents and 50% of the young people completed the treatment satisfaction questionnaire and the findings therefore need to be interpreted with considerable caution. Of those who completed the measure more than 80% of both young people and parents rated their satisfaction with treatment as moderate to high (Table 4). However, the prediction that those who took part in MFT-AN would be more satisfied than those receiving FT-AN was not confirmed either for young people or parents. The parents generally gave higher ratings with nearly 60% being highly satisfied, which was only true of just over 30% of the young people. Comparing parental and young person satisfaction, parental satisfaction was significantly higher than young person’s satisfaction (mean = 2.3; 95% CI 1.16, 3.46; *p* < 0.001). The difference between parental and young person satisfaction was greater for MFT-AN group (mean = 3.19; 95% CI 1.61, 4.76, p < 0.0001) than for FT-AN (mean = 1.39; 95% CI −0.26, 3.04) although this difference between groups did not reach statistical significance (*p* = 0.118).

**Table 4:** Satisfaction with treatment

Client satisfaction questionnaire						
	FT-AN		MFT-AN		Total	
Satisfaction score	Young people	Parents	Young people	Parents	Young people	Parents
8−20 (low)	5 (6.0%)	7 (8.4%)	8 (9.3%)	5 (5.8%)	14 (8.3%)	12 (7.1%)
21−26 (moderate)	19 (22.9%)	13 (15.7%)	21 (24.4%)	15 (17.4%)	39 (23.1%)	28 (16.5%)
27−32 (high)	13 (15.7%)	27 (32.5%)	13 (15.1%)	29 (33.7%)	26 (15.4%)	56 (33.1%)
Missing	46 (55.4%)	36 (43.4%)	44 (51.2%)	37 (43.0)	90 (53.3%)	73 (43.2%)

## Discussion

This is the first randomised trial of multi-family therapy for adolescent anorexia nervosa. The study confirms findings from smaller open studies showing the utility of the multi-family approach [32–35] and adds to the overall evidence for the effectiveness of family therapy in the treatment of adolescent eating disorders [3–5]. The main finding of the study is that while there were clinically significant improvements in both treatments (just under 60% achieving a good or intermediate outcome at the end of treatment in the FT-AN group and more than 75% in the MFT-AN group), there was a statistically significant difference between the two treatment arms in the primary outcome at end of treatment 12 months post baseline. At follow-up (18 months post baseline) there was relatively little change compared to end of treatment although the difference in primary outcome between the two treatments had dropped below the 0.05 level of statistical significance. Significant gains in weight were accompanied by improvements in mood and eating disorder psychopathology but of the secondary outcomes only %mBMI at 18 months showed a statistically significant greater improvement under MFT-AN compared to FT-AN. Approximately half the patients in FT-AN and nearly 60% of those in MFT-AN had started menstruating. Eight young people had some bulimic symptoms at the end of treatment (one of whom would have met a diagnosis of bulimia nervosa) and of these, five still had such symptoms at 18 months. This is not unusual, as the presentation of anorexia nervosa may change either temporarily as part of the process of recovery or as a transition from anorexia nervosa to a bulimia nervosa diagnosis. The number developing BN symptoms in this study (in both treatment arms) is relatively small but we present these details to provide a more comprehensive picture of the recovery process.

Two indicators showed the good acceptability of both treatments – high treatment adherence rates (approximately 90% in each trial arm) and positive ratings on the Client Satisfaction Inventory at the end of treatment. Our prediction that families attending the multi-family treatment would be more satisfied than those receiving singe family therapy was not confirmed but a difference was found between the ratings of parents and young people in both treatments with parents most commonly rating themselves as being highly satisfied whereas the young people were more likely to return ratings of moderately satisfied. While we are cautious about interpreting the treatment satisfaction findings given the low number of returned questionnaires, they are consistent with other studies of family therapy for AN [52]. The good engagement with outpatient treatment is also reflected in only 3% of patients requiring hospital admission during the treatment phase of the study (and less then 7% for the duration of the study as a whole).

Several limitations of the study need to be mentioned. The recruited sample was close to the size required by the power calculation for the purpose of the primary analysis. However, the data collection of secondary outcome variables turned out to be considerably lower than we would have liked and the comparisons on these secondary outcomes therefore has reduced power and need to be considered with caution. Our analyses also do not take account of the fact that MFT-AN was delivered in groups of families, as we had been unable to collect sufficiently reliable information to allow us to account for this in our analysis. However, as only a part of the therapy was delivered in groups with typically 3–4 trial families participating in each group, and the therapist pairs varied across groups we would not expect this to add much variability to the data. The other significant limitations is that the study funding allowed only for the completion of a six month follow-up period after treatment completion and a longer follow would be required to determine the longer term outcomes in the two treatments.

The study also has a number of strengths. It is at present one of the largest RCTs of adolescent AN and because of the relatively few exclusions, with only the most severely ill patients needing urgent hospital admission (those below 67% mBMI or other serious indications of medical instability) being excluded, can be considered largely representative of the clinical population being studied. The pragmatic design of the study meant that the treatments can also be considered to represent the kind of treatment that would be provided in specialist community based ED services in the UK. For instance, therapists were not recruited specifically for the study and included all therapists in the participating services including trainees. While the therapists received additional training and ongoing supervision to deliver the treatments in a manner consistent with the treatment manuals, this is no different from what would be expected in good services purporting to provide evidence-based treatments.

Before drawing conclusions from the study, readers will need to consider several issues. First, the design of the study was such that the amount of treatment was not fully determined by the study protocol but allowed clinicians and the families to decide on the frequency of sessions and this resulted in a somewhat higher amount of therapy time in the MFT-AN arm of the trial and some might argue that this in itself could explain the differences in outcome between the study arms. In order for this to be the case one needs to assume that there is a direct and linear relationship between therapy contact time and outcome, a problematic assumption that takes no account of the fact that change does not happen simply in the context of therapy sessions or that increased frequency of sessions may in some instances inhibit change by fostering a dependence on therapy. In the case of family therapy, which aims to mobilize the family as a resource to promote change, it has long been argued that less frequent sessions particularly in later stages of treatment are often more effective then very frequent meetings as they encourage the family to draw on their own resources which is consistent with the conceptual framework of FT-AN [53]. There is also empirical evidence from research by Lock and colleagues [12, 15] who compared in an RCT 10 and 20 sessions of FT-AN and found no differences in overall outcome with the higher number of sessions suggesting that increasing the “dose” of therapy may not in itself be the key ingredient of change. Nevertheless, the possibility cannot be ruled out that at least some of the differences in outcome between the two treatment arms in the current study were due to increased therapeutic contact with families.

Second, the proportions of those who responded well to the treatment compared to those who had a poor outcome has to be viewed in the context of our choice of outcome criteria. Definitions of what constitutes a positive outcome in anorexia nervosa are as varied as the terms that are used to describe this (good outcome, recovery, remission, partial remission). While nearly all classification of outcome for anorexia nervosa use a weight criterion as a key part of its definition this has varied widely from 85% expected weight to 95% or even 100%. To some extent the lack of agreement on what constitutes a good outcome is simply a reflection of the variability of weight in healthy individuals and the variability in the time lag once healthy weight is achieved and the normalization of physical functions such as menstruation and psychological aspects such as eating disorder cognitions. While in the clinical context we can tolerate the uncertainty of defining how well an individual patient may be doing at a particular point in time, in a research setting we require clear definitions with the consequence that some individuals will be misclassified. The merit of the definition we have chosen is that it has been shown to differentiate outcome between treatments in a number of studies [6, 7] and more importantly that it predicts long term outcome [14, 15]. While it does not provide a direct comparison with studies using a higher weight criterion to define remission, it is readily comparable to definitions of partial remission used in these studies [8, 54].

Nevertheless, comparisons with the results of other studies are not straightforward. Even when comparable definitions of outcome are used, there is considerable variability in the reported outcomes between studies [8, 11, 56]. There are likely to be a number of reasons for this, including differences in demographics, methods of study recruitment and perhaps most importantly differences in healthcare organization between different countries. Thus for instance the UK outpatient RCTs have generally included samples with an average of 75–78% mBMI at baseline [10, 38, 55], whereas in the US and Australian studies baseline weights tend to be higher, ranging on average between 82–86% mBMI [8, 11, 12, 48, 56]. This difference is primarily determined by the way hospital admissions are used in the treatment of anorexia nervosa in different countries. In the US, patients below 75% are generally excluded from outpatient studies (though may be included following a brief admission to increase their weight above this threshold) whereas the UK outpatient studies have generally included patients at lower weights (and only admitted to hospital if other criteria of medical instability are present). Similarly, in the US and Australian studies the number of hospital admissions during trials tend to be higher than in the UK although these admissions are generally very brief. In the UK studies while admissions are mostly less frequent they tend to be longer. We do not know what effect these differences are likely to have on the process of recovery and the weight that patients reach at the end of treatment which not only makes comparisons with other studies difficult but also limits the generalizability of the study findings from one health service context to another [57].

Treatment outcome is also likely to be influenced by other factors such duration of illness [58]. The duration of illness in the present study is comparable to most of the published adolescent AN RCTs and is also consistent with service level research in the UK [57, 59]. However, a recent study in Germany found that the duration of illness before first treatment of young people with AN in a number of different treatment settings was nearly 2–3 times as long [60], again highlighting the need for caution in generalizing findings across different healthcare settings.

## Conclusions

The outcome results of the current study in both treatments but particularly the MFT-AN arm compare well with other adolescent RCTs, especially considering the low baseline weights of the study participants. However, given the above caveats in mind and the fact that this is still the only RCT of MFT-AN we are cautious in our conclusions. Moreover, the study inevitably raises many new questions. In this study the MFT-AN trial arm combined single and multi family sessions reflecting the way in which MFT-AN has been developed at the Maudsley Hospital. Other services have in the mean time applied the MFT model in different ways using MFT-AN as a standalone outpatient treatment [32, 34, 36] or as part of day care [33] or inpatient treatment [61]. Our study included only young people with a diagnosis of AN or restricting EDNOS (again reflecting the current clinical practice at the Maudsley service) while others have utilised the multifamily interventions with mixed ED diagnoses groups. In a recent paper [62], we have described MFT aimed specifically at adolescents suffering from BN, which is less intensive than MFT-AN and includes CBT and DBT components in the treatment. The current study shows that the particular combination of intensive MFT-AN with ongoing single FT-AN and MFT-AN meetings improves outcomes at end of treatment compared to FT-AN and highlights the benefits of bringing groups of families together as a potentially powerful treatment resource. A great deal more research is needed, however, to identify how this resource can best be utilised in different clinical contexts and with different ED clinical groups.

## Abbreviations

%mBMI: Percent median BMI
AN: Anorexia nervosa
BDI: Beck Depression Inventory
BMI: Body mass index
BN: Bulimia nervosa
CBT: Cognitive behavioural therapy
CI: Confidence interval
CSQ: Client Satisfaction Questionnaire
DBT: Dialectical behaviour therapy
DSM: Diagnostic and statistic manual of mental disorders
ECI: Experience of Caregiving Inventory
ED: Eating disorder
EDE: Eating Disorder Examination
FBT: Family based treatment
FT: Family therapy
MFT: Multi-family therapy
MR: Morgan Russell scale
OCD: Obsessive, compulsive disorder
OR: Odds ratio
RCT: Randomised controlled trial
RSE: Rosenberg Self Esteem scale

## Footnotes

1 Various labels have been used for eating disorder focussed family therapy including Maudsley Family Therapy, Maudsley Model Therapy and in recent years most commonly Family Based Treatment or FBT. We have argued [19] that such terms are misleading because they imply a theoretically distinct model of treatment rather than a treatment with a shared theoretical frame with other systemic therapies but with a specific focus on treating an eating disorder (very much in the way that “anxiety focussed CBT” or “trauma focussed CBT” are both forms of CBT). The term FBT is also ambiguous because it is sometimes used just to refer to treatments using a specific treatment manual [63] and sometimes to all studies using an eating disorders focussed family therapy. Our use of the term FT-AN emphasizes that this is a treatment that draws on family systems theory and uses a broad range of family therapy intervention techniques while its specificity derives from its focus on treating an eating disorder [64].

2 While most adolescent studies have used 18 years as their upper age limit we decided to include a broader age range for two main reasons. First, 18 years is an arbitrary boundary that reflects some changes in a young person’s legal status but certainly does not reflect either a social or biological maturational boundary between adolescence and adulthood. Second, there is increasing recognition that transitions between adolescent and adult services need to be more flexible and we therefore wanted to make sure that our findings would be applicable beyond the narrowly defined boundary between services.
